# mGlu3 receptor regulates microglial cell reactivity in neonatal rats

**DOI:** 10.1186/s12974-020-02049-z

**Published:** 2021-01-06

**Authors:** Manuela Zinni, Jérôme Mairesse, Julien Pansiot, Francesco Fazio, Luisa Iacovelli, Nico Antenucci, Rosamaria Orlando, Ferdinando Nicoletti, Daniel Vaiman, Olivier Baud

**Affiliations:** 1 Inserm UMR1141 NeuroDiderot, Univ. Paris Diderot, Sorbonne Paris Cité, Paris, France; 2 Laboratory of Child Growth and Development, University of Geneva, Geneva, Switzerland; 3 IRCCS Neuromed, Pozzilli, Italy; 4 Department of Physiology and Pharmacology, Sapienza University of Rome, Rome, Italy; 5 Institut Cochin, Inserm U1016, UMR8104 CNRS, Paris, France; 6 Division of Neonatology and Pediatric Intensive Care, Children’s University Hospital of Geneva, Geneva, Switzerland

**Keywords:** mGlu3 receptor, Microglia reactivity, Perinatal neuroinflammation

## Abstract

**Background:**

Perinatal inflammation is a key factor of brain vulnerability in neonates born preterm or with intra-uterine growth restriction (IUGR), two leading conditions associated with brain injury and responsible for neurocognitive and behavioral disorders. Systemic inflammation is recognized to activate microglia, known to be the critical modulators of brain vulnerability. Although some evidence supports a role for metabotropic glutamate receptor 3 (mGlu3 receptor) in modulation of neuroinflammation, its functions are still unknown in the developing microglia.

**Methods:**

We used a double-hit rat model of perinatal brain injury induced by a gestational low-protein diet combined with interleukin-1β injections (LPD/IL-1β), mimicking both IUGR and prematurity-related inflammation. The effect of LPD/IL-1β on mGlu3 receptor expression and the effect of mGlu3 receptor modulation on microglial reactivity were investigated using a combination of pharmacological, histological, and molecular and genetic approaches.

**Results:**

Exposure to LPD/IL-1β significantly downregulated Grm3 gene expression in the developing microglia. Both transcriptomic analyses and pharmacological modulation of mGlu3 receptor demonstrated its central role in the control of inflammation in resting and activated microglia. Microglia reactivity to inflammatory challenge induced by LPD/IL-1β exposure was reduced by an mGlu3 receptor agonist. Conversely, both specific pharmacological blockade, siRNA knock-down, and genetic knock-out of mGlu3 receptors mimicked the pro-inflammatory phenotype observed in microglial cells exposed to LPD/IL-1β.

**Conclusions:**

Overall, these data show that Grm3 plays a central role in the regulation of microglial reactivity in the immature brain. Selective pharmacological activation of mGlu3 receptors may prevent inflammatory-induced perinatal brain injury.

## Background

Every year, 30 million infants worldwide are delivered after intra-uterine growth restriction (IUGR), and 15 million are born preterm [1, 2]. These two complications are the leading causes of ante/perinatal stress and brain injury, responsible for neurocognitive and behavioral disorders in more than 9 million children each year. Both prematurity and IUGR are associated with perinatal systemic inflammation, identified to activate microglia [3], the resident macrophages of the central nervous system (CNS), and to be the best predictor of subsequent neurological impairment [4, 5]. Microglial cells can acquire distinct phenotypes in response to perinatal stimuli that allow them to not only disrupt developmental processes but also support repair and regeneration. Recently, it was hypothesized that the loss of control of postnatal microglial activation is a precipitating factor for neurodevelopmental disorders from cerebral palsy to autism spectrum disorders [6]. The control of microglial activation during this critical period therefore appears to be key in protecting the developing brain.

The level of glutamate, the major excitatory neurotransmitter in the CNS, is finely tuned during the early postnatal period, when the developing brain is highly vulnerable to excitotoxic insults. The action of glutamate on microglia in perinatal life is largely unexplored. Indeed, most studies focusing on the roles of glutamate during the neonatal period have explored neuronal and astroglial cell function [7–13].

Metabotropic glutamate (mGlu) receptors form a family of eight subtypes, subdivided into three groups on the basis of their amino acid sequence, pharmacological profile, and G protein coupling [14]. Their dysfunction is commonly observed in the pathophysiology of neurodegenerative and neuropsychiatric diseases [15–19]. They are expressed in mature microglia and modulate microglial functions in physiology and pathology [20–23]. In particular, mGlu3 and mGlu5 have shown to be the predominant mGlu receptors expressed in glial cells both in vivo and in vitro [24–26]. Thompson et al. showed that activation of microglial mGlu2 and mGlu3 promotes a neurotoxic microglial phenotype [27].

Targeting mGlu receptors represents a realistic therapeutic strategy due to the availability of allosteric modulators with long-lasting effects and a better safety profile than pharmacological agents that modulate ionotropic glutamate receptors. Indeed, recent evidence shows positive allosteric modulators of mGlu3 receptors in astrocytes or mGlu4 receptors in oligodendrocytes to be promising pharmacological approaches for the treatment of neurodegenerative disorders [28].

The only published study concerning neonatal microglia reported a role for mGlu5 receptors in the control of neuroinflammation following excitotoxic insult [7]. Recent studies have shown that mGlu3 receptors boost mGlu5 receptor signaling in brain tissue and cultured astrocytes and that mGlu3 activation is required for a full mGlu5 receptor response to agonist activation [29–31]. We therefore investigated whether mGlu3 receptors, encoded by Grm3 gene, play a role in the modulation of brain inflammation, in particular in microglial cell reactivity.

We investigated Grm3 gene expression in vivo and in primary microglial cells sorted from neonatal rats. Bioinformatics analysis of a large microarray dataset shows Grm3 to be a major hub in the control of inflammation in resting and activated microglia. We also show that targeting mGlu3 receptors drastically modulates microglial activation using pharmacological, molecular, and genetic approaches.

## Materials and methods

### Animals, diets, and postnatal inflammatory challenge: LPD/IL-1β rat model

All experiments were carried out in compliance with Inserm ethical rules, approved by the institutional review board (Ministry of Higher Education and Scientific Research, Directorate-General for Research and Innovation, Paris, France), in accordance with the European Communities Council Directive 2010/63/EU. Briefly, Sprague-Dawley dams (Janvier Labs, Le Genest-Saint-Isle, France) were randomly divided into two groups according to their diet: a 22% (normal) protein diet (control, CTRL) or an isocaloric 9% protein diet (LPD) from the day of conception until delivery. On postnatal day 1 (P1) and P2, pups were injected intraperitoneally (i.p.), twice a day, with PBS or IL-1β (20 μg/kg, Miltenyi Biotec, Bergisch Gladbach, Germany), as previously reported [32].

### Postnatal inflammatory challenge in Grm3^−/−^ mice

All experiments were carried out in compliance with the local ethical committee of IRCCS Neuromed (OPBA) approved by the Italian Ministry of Health in accordance with the European Communities Council Directive 2010/63/EU. We used adult mGlu3 receptor knock-out mice and their C57BL/6 J wild type (WT) counterparts bred at IRCCS Neuromed (Pozzilli, Italy). Grm3^−/−^ mice were originally generated by targeted disruption of exon II containing the open reading frame of the Grm3 gene as described previously [33]. Fetal loss was observed in mice exposed to gestational LPD. Hence, response of Grm3^−/−^ and Grm3^+/+^ (WT) pups generated by homozygous breeding to systemic inflammation was studied in animals treated i.p. at P1 with IL-1β (10 μg/kg, Miltenyi Biotec, Bergisch Gladbach, Germany) every 12 h for 3 days, and compared to PBS-injected animals. Mice were euthanized at P4 (12 h after the last injection), and the brains were collected and separated in the two hemispheres. One hemisphere was used for Iba1 immunolabeling whereas the other was used for cytokine cortical expression analysis using real-time quantitative PCR (RT-qPCR).

### Cortical sample collection and magnetic sorting of microglial cells

Cortical samples were collected from CTRL and LPD/IL-1β pups at P1, P4, P7, P10, and P20. Primary microglial cells were sorted at P1, P4, P7, P10, and P20 using magnetic antibody-based cell sorting (MACS) and CD11b antibody (Miltenyi Biotec, Bergisch Gladbach, Germany), as previously reported [32]. The purity of the P4 and P7 MACS microglial cell sorting was validated by RT-qPCR performed on the positive and negative CD11b+ cell fractions for Itgam, Gfap, Neun, and Mbp and the arbitrary units normalized to the respective negative fraction in CTRL: P4 CTRL (mean ± SEM)—Itgam 99.78 ± 11.29, Gfap 0.13 ± 0.01, Neun 0.08 ± 0.02, and Mbp 0.02 ± 0.003; P4 LPD/IL-1β—Itgam 109.15 ± 5.27, Gfap 0.11 ± 0.1, Neun 0.02 ± 0.002, and Mbp 0.01 ± 0.01; P7 CTRL—Itgam 203.80 ± 9.95, Gfap 0.07 ± 0.01, Neun 0.04 ± 0.004, and Mbp 0.06 ± 0.02; and P7 LPD/IL-1β—Itgam 277.5 8 ± 23.27, Gfap 0.09 ± 0.01, Neun 0.05 ± 0.01, and Mbp 0.02 ± 0.003.

### RNA purification, cDNA synthesis, and real-time qPCR

Samples were immediately snap frozen after collection. Total RNA from the cortex was extracted using Nucleazol reagent and the NucleoSpin RNA Set for NucleoZol (Macherey-Nagel, Hœrdt, France). Microglial RNA was isolated using NucleoSpin RNA Plus XS (Macherey-Nagel). RNA quantity and quality were determined using the NanodropTM apparatus (Thermofisher Scientific, Waltham, MA, USA). Reverse transcription was performed using the IscriptTM cDNA synthesis kit (Bio-Rad, Marnes-la-Coquette, France). Primers were designed using Primer3Plus software, and sequences are available on request. RT-qPCR was performed in triplicate as reported in [34] using the Ribosomal protein L13 (Rpl13) as the reference gene.

### RNA preparation from sorted microglial cells, cDNA synthesis, microarray hybridization, and bioinformatics analysis

Total RNA was extracted from CTRL and LPD/IL-1β P4 animals, and cDNA synthesis and microarray hybridization performed as previously described [32]. For microarray hybridization, labeled cRNA was generated using classical protocols for Affymetrix array hybridization. Three points per condition were analyzed by RaGene-2_0-st microarray hybridization, for which 36,685 probes are examined and directly analyzed using Gene Set Enrichment Analysis (https://www.gsea-msigdb.org/gsea/index.jsp [35]). Network analysis of the microarray data was also performed using String (https://string-db.org/). The numerical data from the networks obtained were then exported to Cytoscape (https://cytoscape.org/) to identify putative hub genes, using the Network Analysis option. Microarray results were validated as previously reported [32].

### Protein extraction and immunoblotting assay

Total protein was extracted from the cortex and microglial cells sorted from P4 and P7 rats of the CTRL and LPD/IL-1β experimental groups. Cortex samples were homogenized in lysis buffer (0.32 M sucrose, 4 mM HEPES pH 7.4, 1% SDS). Complete, Mini, EDTA-free Protease Inhibitor Cocktail and PhosSTOP (Roche, Meylan, France) were added. RIPA buffer (Sigma-Aldrich, St Quentin Fallavier, France), supplemented with protease and phosphatase inhibitors, was used to extract protein from the microglial cells. Proteins were quantified using the Bradford assay (Sigma-Aldrich) and 30 μg from the cortex and 20 μg from the microglia re-suspended in Laemmli Sample Buffer (Bio-Rad) with 2.5% 2-mercaptoethanol (Sigma-Aldrich). Samples were separated on 4–15% Mini-PROTEAN TGX Precast Protein Gels and the proteins transferred to nitrocellulose membranes using a Trans-Blot Turbo Mini (Bio-Rad). Proteins were stained using Ponceau S solution (Sigma-Aldrich) and digital images acquired. The optical density of each well was measured using NIH ImageJ medical imaging software. Blots were incubated in a blocking solution containing Tris-buffered saline (TBS), 0.1% Tween-20 (Sigma-Aldrich), and 5% non-fat milk (Bio-Rad) for 1 h at room temperature. Blots were then incubated overnight with rabbit anti-mGlu3 receptor (1:600; AGC-012, Alomone Labs, Jerusalem, Israël) in blocking solution at 4 °C. After incubation with the primary antibody, the blots were incubated with horseradish peroxidase-conjugated goat anti-rabbit (1:2000; 0545, Sigma-Aldrich) for 1 h at room temperature. Bands were visualized by enhanced chemiluminescence using Clarity Max ECL (Bio-Rad). Digital images were acquired and the optical density of each band measured as described above. The ratio of the target to Ponceau S was then determined and the values compared for statistical significance.

### Drugs

LY 379268, a highly selective group II mGlu receptor agonist (Tocris Bioscience, Rennes, France), was dissolved in sterile water. LY 2389575 hydrochloride, a selective negative allosteric modulator of mGlu3 receptors (Tocris), and Ro 64-5229, a selective, non-competitive mGlu2 receptor antagonist (Tocris), were dissolved in DMSO.

### Primary microglial cell culture: real-time qPCR and morphological assay

Microglial cells were isolated from CTRL and LPD/IL-1β animals at P4 and P7 and cultured as previously reported [32]. CTRL microglial cells were treated with LY 379268 (0.1, 0.3, 1, 3, or 5 μM) + Ro 64-5229 (25 μM) to establish the optimal drug concentration (1 μM). After 48 h, CTRL and LPD/IL-1β microglia were divided into four groups: (1) DMSO (0.30%), (2) LY 379268 (1 μM) + Ro 64-5229 (25 μM), (3) IL-1β (50 ng/ml) + IFNγ (20 ng/ml) for 1 h, and (4) LY 379268 (1 μM) + Ro 64-5229 (25 μM) for 1 h and then IL-1β + IFNγ for 4 h.

A second series of experiments was performed on P4 and P7 CTRL microglial cells. CTRL microglial cells were treated with LY 2389575 (0.1, 0.3, 1, 3, or 5 μM) to establish the optimal drug concentration (5 μM). Forty-eight hours after plating, cells were divided into four groups: (1) DMSO (0.30%), (2) LY 2389575 (5 μM), (3) IL-1β + IFNγ for 1 h, and (4) LY 2389575 (5 μM) for 1 h and then IL-1β + IFNγ for 4 h.

Total RNA was extracted using NucleoSpin RNA Plus XS. RNA quality and quantity were determined, cDNA synthesized, and real-time qPCR performed as reported above.

Morphological analysis was performed after increasing the drug exposure time to 12 h. After fixation with 4% PFA, cells were stained with goat anti-Iba1 (1:500; ab5076, Abcam, Paris, France) and DAPI (1:10,000). Iba1^+^ cells were analyzed using a fluorescent microscope (Nikon Eclipse Ti-E): (i) area, (ii) perimeter, and (iii) cell circularity (4π × (area/perimeter^2^)) were determined.

### Immunofluorescence

Brains were collected from WT and Grm3^−/−^ pups at P4, embedded in paraffin, and cut coronally in 10-μm-thick slices. Paraffin-embedded sections were immunolabeled with goat anti-Iba1 (1:500; ab5076, Abcam, Paris, France) and DAPI (1:10,000) and labeling visualized using the secondary antibodies coupled to the red fluorescent marker Cyanine 3 (Life technology) as previously described [30]. Stained sections were mounted on microscope slides with Fluoromount G (Southern Biotech). Three slices per animal were analyzed using a fluorescent microscope (Nikon Eclipse Ti-E): (i) area, (ii) perimeter, and (iii) cell circularity (4π × (area/perimeter^2^)) were determined.

### siRNA experiments

Experiments to knock-down Grm3 gene expression were performed on P7 CTRL microglia using Viromer Blue Reagent (Lipocalyx, Halle, Germany) according to the manufacturer’s protocol. Transfection efficiency and cell viability after Viromer exposure were evaluated using the siGLO Red Transfection Indicator (25 nM or 50 nM) (Dharmacon, Lafayette, CO, USA). Cells were fixed in 4% PFA, stained with anti-Iba1 antibody, and analyzed by fluorescence microscopy. Cells were treated 48 h after plating with (1) ON-TARGETplus Non-targeting Pool (25 nM or 50 nM) or (2) ON-TARGETplus Rat Grm3 (24416) siRNA - SMARTpool (25 nM or 50 nM) (Dharmacon) for 4 h. The medium was replaced by fresh culture medium, and the cells incubated at 37 °C for 48 h before proceeding to RNA and protein extraction.

### Statistical analysis

Statistical analysis of all data was performed using GraphPad PRISM version 8.0 (San Diego, CA, USA). Student’s *t* test was performed for two-group comparisons. A one- or two-way ANOVA, followed by the Newman-Keuls post hoc multiple comparison tests, was performed for comparison of more than two groups. A Pearson correlation test was used to analyze relationship between Grm3 and inflammatory cytokines’ mRNA expression in siRNA experiment. A multiple *t* test was performed to analyzed microarray data. Significance was set at *p* < 0.05 for all tests. Number of samples, statistics, and *p* values are reported in Additional Table 1. Morphological data and Iba1^+^ cell density were analyzed by a person who was blind to experimental conditions. Pups from 2–3 litters were used in each experiment.

## Results

### LPD/IL-1β exposure in rats induces downregulation of Grm3 gene expression in microglia

**Fig. 1:**
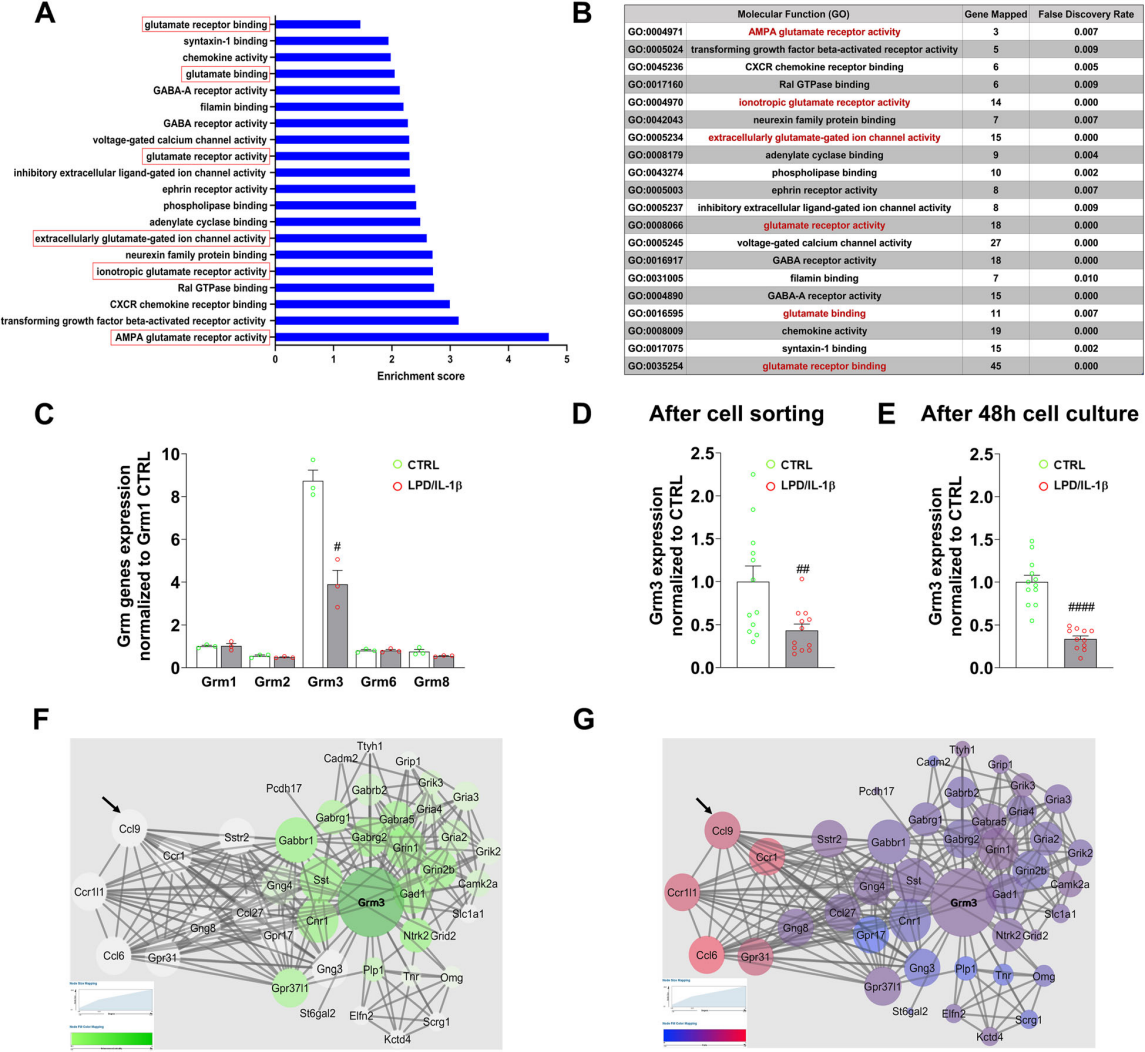
Transcriptomic analysis of Grm3 deregulation associated with neonatal neuroinflammation in rats. **a** Functional network enrichment in microglial cells magnetically sorted from P4 animals under basal conditions (CTRL) and following LPD/IL-1β challenge inducing neonatal neuroinflammation. The 20 most enriched molecular functions are reported in the graph, sorted by increasing enrichment score. The ontologies associated with glutamate receptor activity are underlined in red. **b** Description of the number of genes mapped and their respective FDR values for each molecular function. In red are reported the gene ontology pathways that were associated with glutamate receptor activity. **c** Grm gene expression in microglia cells sorted from CTRL and LPD/IL-1β animals at P4 according to a Gene Set Enrichment Analysis against the Gene Ontology Database. Data (mean ± SEM) are relative to Grm1 expression in CTRL animals. Multiple *t* test; ^#^*p* < 0.05. **d** Grm3 gene expression in microglial cells just sorted from CTRL and LPD/IL-1β animals at P4 analyzed by quantitative RT-PCR. Data (mean ± SEM) are relative to Grm3 expression in CTRL animals. Unpaired *t* test; ^##^*p* < 0.01. **e** Grm3 gene expression assessed in primary microglia from CTRL and LPD/IL-1β animals at P4 after 48 h cell culture. Data (mean ± SEM) are relative to Grm3 expression in CTRL animals. Unpaired *t* test; ^####^*p* < 0.0001. **f** Minimal Grm3 network showing the centrality of Grm3 gene deregulation following LPD/IL-1β exposure, selecting deregulated genes that are connected to more than 10 genes. Betweenness centrality of hub genes is quantified by the circle size, and the number of connections among genes by the color code. The figure shows that Grm3 has a central position in the network. **g** The same Grm3 minimal network as in **f** showing the expression of Grm3 and Grm3-associated genes. The blue color vs the red color intensity is associated with down- and upregulation, respectively

We studied the transcriptomic consequences of the combination of gestational LPD and neonatal IL-1β exposure on the glutamatergic system in microglial cells. Bioinformatics analysis performed on microglial cells sorted from P4 rat pups showed consistent significant alterations of the glutamatergic functions in animals subjected to LPD/IL-1β relative to CTRL animals (Fig. 1a). Overall, gene sets related to both glutamate-receptor binding and activity were significantly enriched in LPD/IL-1β-exposed animals (Fig. 1a, b). Focusing on metabotropic receptors, the microarray analysis showed the mGlu3 receptor-coding gene (Grm3) to be one of the most highly expressed mGlu receptors at P4 in primary microglial cells and the most deregulated among group II mGlu receptor-coding genes (Fig. 1c). Grm3 was downregulated in sorted microglia, both before plating (Fig. 1d) and after 2 days of cell culture (Fig. 1e). Network analysis showed that Grm3 is a major “hub” gene in a 43-gene network (Fig. 1f) and that the network centered on Grm3 is connected to another network “Cytokine-cytokine receptor interaction/Chemokine signaling pathway,” characterized by the genes Ccl9, Ccl6, Ccr1, and Ccr1l1. Analysis of gene expression within the same network showed Grm3 downregulation to be consistently associated with upregulation of these inflammation-related genes (Fig. 1g).

**Fig. 2:**
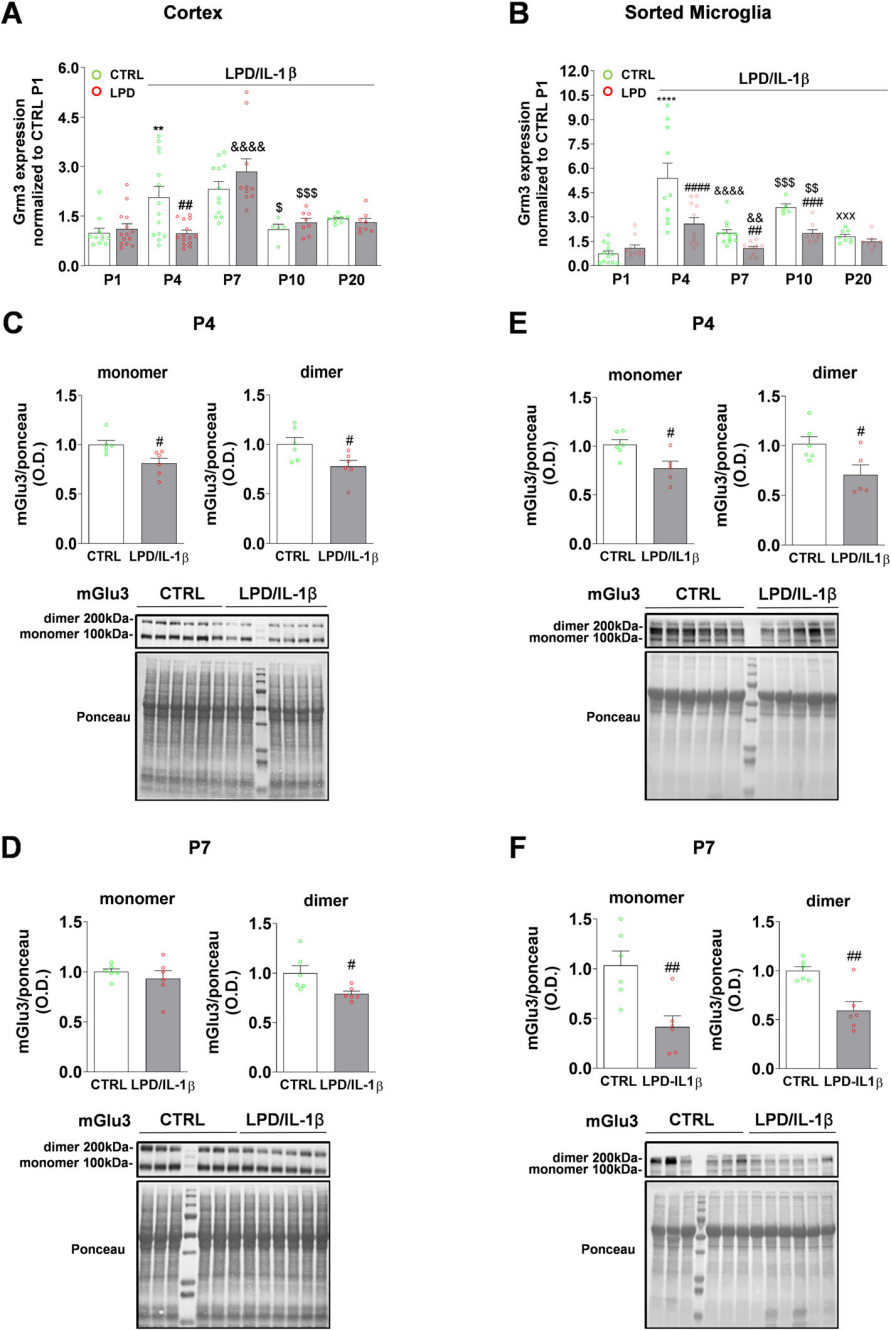
Developmental expression of mGlu3 receptors following the LPD/IL-1β double hit in rat. **a**, **b** Grm3 mRNA expression in cortex (**a**) and in microglia (**b**) sorted from CTRL and LPD/IL-1β animals. Data (mean ± SEM) are relative to Grm3 expression in sorted cells of CTRL cortex and CTRL microglia at PND1. Two-way ANOVA followed by the Newman-Keuls multiple comparison test; **p < 0.01, ****p < 0.0001 vs respective P1; ^&&^*p* < 0.01, ^&&&&^*p* < 0.0001 vs respective P4; ^$$^*p* < 0.01, ^$$$^*p* < 0.001 vs respective P7; ^xxx^*p* < 0.001 vs respective P10; ^#^*p* < 0.05, ^##^*p* < 0.01, ^###^*p* < 0.001, ^####^*p* < 0.0001 effect of LPD/IL-1β. **c**–**f** mGlu3 protein quantification in cortex and primary microglia cells at P4 (**c**, **e**) and P7 (**d**, **f**) in CTRL and LPD/IL-1β pups. P4 cortex and microglia cells sorted from three animals were pooled. Data (mean ± SEM). Quantification of mGlu3 receptor expression in tissue or cells from LPD/IL-1β pups was performed relative to that of CTRL pups. The optical density (O.D.) for mGlu3 receptors was normalized to the O.D. of Ponceau S staining. Unpaired *t* test; ^#^*p* < 0.05, ^##^*p* < 0.01

We next assessed whether the developmentally regulated expression of Grm3 during the first three postnatal weeks was altered by LPD/IL-1β exposure. RT-qPCR analysis showed that Grm3 expression in the cerebral cortex increased from birth to P4/P7, followed by a decline from P10 in CTRL animals. The early postnatal increase in Grm3 expression was delayed in rats exposed to LPD/IL-1β, showing significantly lower expression of Grm3 at P4 than in CTRL animals (Fig. 2a).

This developmental profile was also observed in microglial cells, with maximum expression at P4 and a long-lasting reduction in Grm3 levels between P4 and P10 in animals exposed to LPD/IL-1β (Fig. 2b). Alterations of Grm3 gene expression induced by LPD/IL-1β exposure were further confirmed by immunoblot analysis, showing a significant reduction at P4 in both the cortex (Fig. 2c) and sorted microglia (Fig. 2e). We also observed this reduction at P7 (Fig. 2d, f).

### In vitro mGlu3 receptor activation limits microglial reactivity following LPD/IL-1β exposure in rats

Microglial cells sorted from CTRL rats were stimulated with IL-1β and IFNγ and treated with both the mGlu2/3 receptor agonist LY 379268 and Ro 64-5229, a selective non-competitive mGlu2 receptor antagonist, to assess the effect of mGlu3 receptor activation on inflammatory-related gene expression. Addition of IL-1β + IFNγ caused a highly significant increase in pro-inflammatory cytokines’ gene expression and, to a lesser extent, in anti-inflammatory and immunoregulatory cytokines’ gene expression. Selective activation of mGlu3 receptors by LY 379268 (1 μM) largely reduced the transcription of Il6, Tnfa, and Nos2 genes (Additional Fig 1A), without significant change in the expression of anti-inflammatory and immunoregulatory cytokines (Additional Fig 1B).

**Fig. 3:**
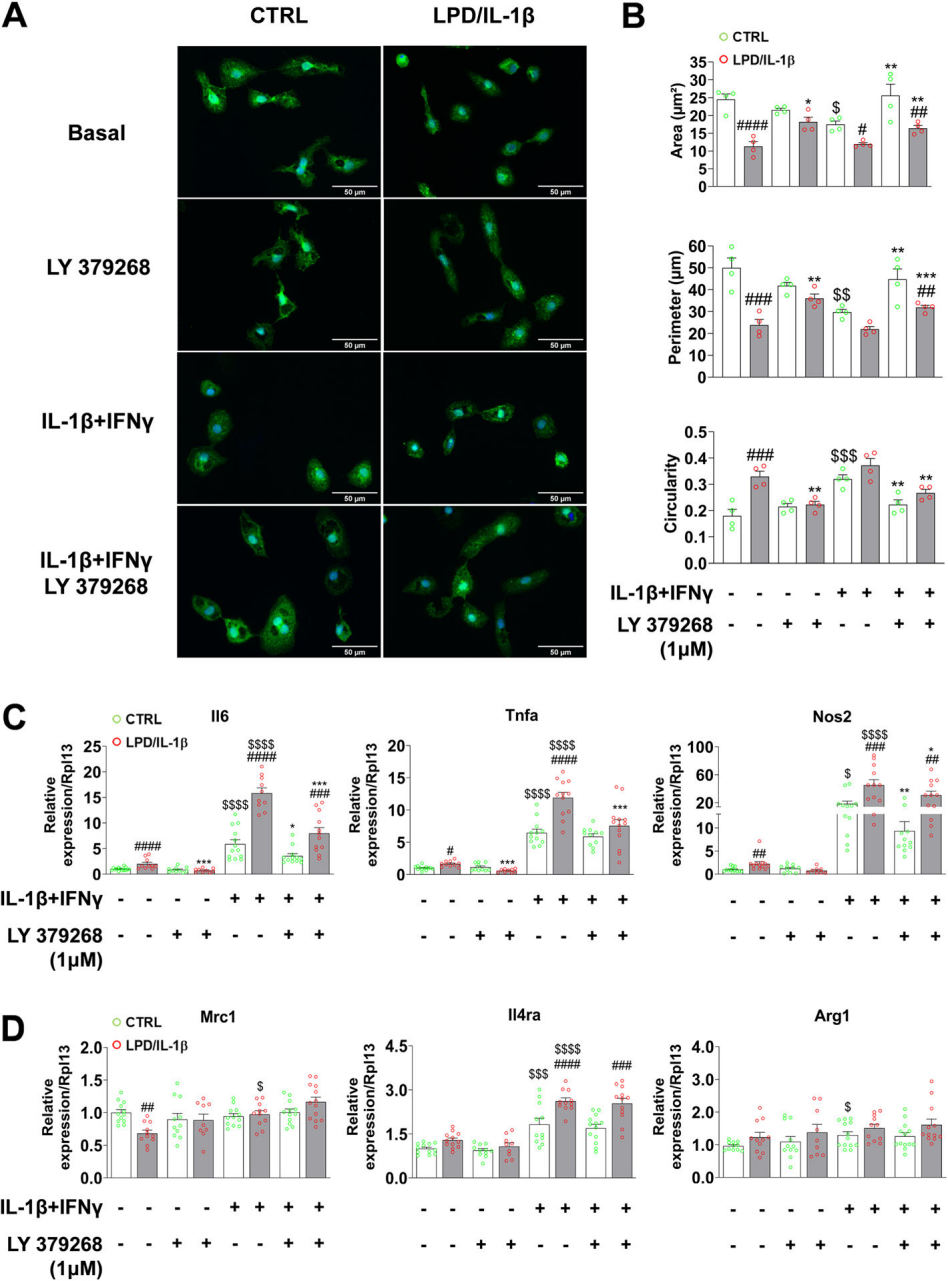
mGlu3 receptor activation and microglial reactivity in CTRL and LPD/IL-1β cultured microglia at P4. **a**, **b** Microglial cells were stained with Iba1 (green) and DAPI (blue) under basal and challenged conditions (IL-1β + IFNγ) ± LY 379268 (1 μM) + Ro 64-5229 (25 μM). Representative photomicrographs at × 40 magnification are shown in **a** (scale bar = 50 μm). Four cell-culture wells for each condition were analyzed (mean cell number, 165 ± 1 7) and the cell area, cell perimeter, and cell circularity were assessed in **b**. Data (mean ± SEM). Two-way ANOVA followed by the Newman-Keuls multiple comparison test; *p < 0.05, **p < 0.01, ***p < 0.001 effect of LY379268 + Ro 64-5229; ^$^*p* < 0.05, ^$$^*p* < 0.01, ^$$$^*p* < 0.001 effect of IL-1β + IFNγ; ^#^*p* < 0.05, ^##^*p* < 0.01, ^###^*p* < 0.001, ^####^*p* < 0.0001 effect of LPD/IL-1β. **c**, **d** mRNA expression of pro-inflammatory (**c**) and anti-inflammatory/immune-regulatory (**d**) markers under basal and pro-inflammatory conditions ± LY 379268 (1 μM) + Ro 64-5229 (25 μM). Data (mean ± SEM) are relative to the gene expression under basal CTRL conditions. Two-way ANOVA followed by the Newman-Keuls multiple comparison; *p < 0.05, **p < 0.01, ***p < 0.001 effect of LY 379268 + Ro 64-5229; ^##^*p* < 0.01, ^###^*p* < 0.001, ^####^*p* < 0.0001 effect of LPD/IL-1β; ^$^*p* < 0.05, ^$$$^*p* < 0.001; ^$$$$^*p* < 0.0001 effect of IL-1β + IFNγ

We next assessed the effect of pharmacological activation of mGlu3 receptors on microglial reactivity in cultured microglia sorted from P4 rats that had been exposed to LPD/IL-1β and age-matched CTRL rats. In vitro, LPD/IL-1β induced changes in the morphology of cultured microglia, which showed an amoeboid shape, characterized by a reduced area, reduced perimeter, and increased circularity (Fig. 3a, b). In vitro inflammatory challenge with IL-1β + IFNγ induced changes in the cell morphology of microglial cells sorted from CTRL animals but not that of cells sorted from animals exposed to LPD/IL-1β, which were already activated. The constitutive inflammatory phenotype observed in microglia sorted from rats exposed to LPD/IL-1β was reversed by mGlu3 receptor activation. Morphological changes induced by in vitro stimulation with IL-1β + IFNγ were also partially prevented by mGlu3 receptor activation (Fig. 3a, b).

We next examined the effect of mGlu3 receptor activation on the expression of inflammatory markers in microglia sorted from pups exposed to LPD/IL-1β or control microglia, with or without in vitro stimulation with IL-1β + IFNγ. Microglia from LPD/IL-1β-exposed animals showed high expression of Il6, Tnfa, and Nos2 (Fig. 3c) and lower expression of Mrc1 (Fig. 3d) under basal conditions. Stimulation with IL-1β + IFNγ induced a greater increase in inflammatory marker expression in microglia sorted from LPD/IL-1β rats than in microglia from CTRL rats (Fig. 3c, d). Pharmacological activation of mGlu3 receptors mitigated the basal higher reactivity of microglia sorted from P4 animals exposed to LPD/IL-1β and also reduced the pro-inflammatory effect of IL-1β + IFNγ challenge in microglia sorted from both CTRL and LPD/IL-1β-exposed animals (Fig. 3c).

We performed similar experiments using cultured microglia sorted from P7 animals, in which cells sorted from animals exposed to LPD/IL-1β showed an activated phenotype under basal conditions and a greater pro-inflammatory response to in vitro challenge with IL-1β + IFNγ (Additional Fig 2A, B, C). These changes were all reversed or mitigated by pharmacological activation of mGlu3 receptors.

### In vitro pharmacological blockade of mGlu3 receptors induces a pro-inflammatory phenotype in microglia sorted from rat pups

It was important to demonstrate that endogenous blockade of mGlu3 can replicate the pro-inflammatory phenotype observed in microglia sorted from rats exposed to LPD/IL-1β in microglia sorted from control pups. These experiments are key to validate mGlu3 receptors as candidate drug targets to alleviate perinatal neuroinflammation associated with IUGR or prematurity. Our first strategy was to use compound LY 2389575, a negative allosteric modulator of mGlu3 receptors [36]. Selective pharmacological blockade of mGlu3 receptors using LY 2389575 exacerbated the effect of in vitro inflammatory challenge with IL-1β + IFNγ on pro-inflammatory cytokines’ gene expression (Additional Fig 3A) and conversely significantly reduced the gene expression of the anti-inflammatory cytokine Arg1 (Additional Fig 3B).

**Fig. 4:**
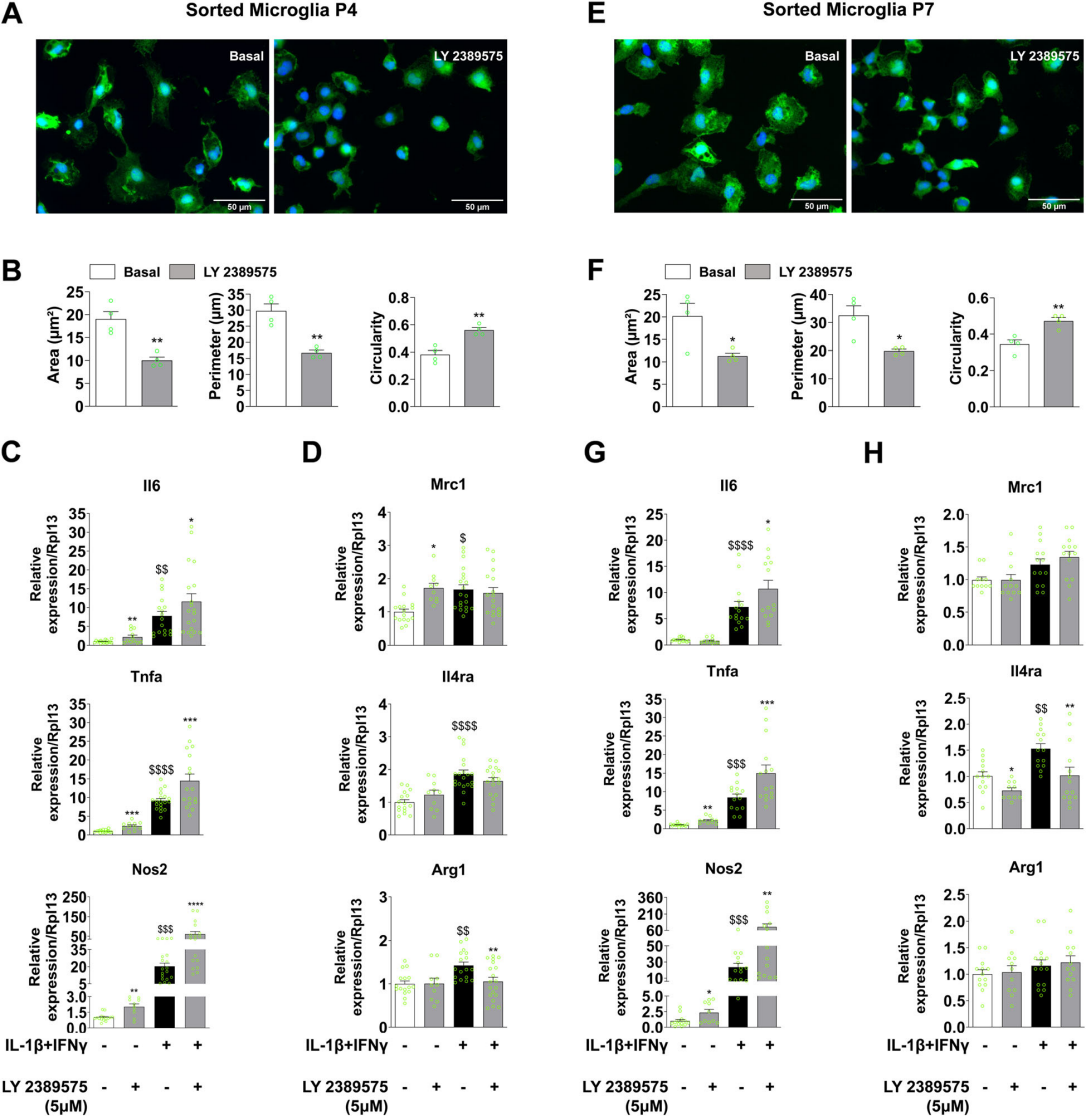
Pharmacological mGlu3 receptor blockade and microglial reactivity in response to inflammatory stimulation in vitro. **a**, **b** Microglial cells sorted from P4 pups were stained with Iba1 (green) and DAPI (blue) under basal conditions ± LY 2389575 (5 μM). Representative photomicrographs at × 40 magnification are shown in **a** (scale bar = 50 μm). The cell area, cell perimeter, and cell circularity were assessed in **b** (mean cell number, 162 ± 10). Data (mean ± SEM). Unpaired *t* test; **p < 0.01. **c**, **d** mRNA expression of pro-inflammatory (**c**) and anti-inflammatory/immune-regulatory (**d**) markers in microglial cells sorted from P4 pups cultured under basal or pro-inflammatory (IL-1β + INFγ) conditions ± LY 2389575 (5 μM). The data (mean ± SEM) are relative to the gene expression under basal CTRL conditions. One-way ANOVA followed by the Newman-Keuls multiple comparison test; *p < 0.05, **p < 0.01, ***p < 0.001, ****p < 0.001 effect of LY 2389575; ^$^*p* < 0.05, ^$$^*p* < 0.01, ^$$$^*p* < 0.001, ^$$$$^*p* < 0.0001 effect of IL-1β + INFγ. **e**, **f** Microglial cells sorted from P7 pups were stained with Iba1 (green) and DAPI (blue) under basal conditions ± LY 2389575 (5 μM). Representative photomicrographs at × 40 magnification are shown in **e** (scale bar = 50 μm). The cell area, cell perimeter, and cell circularity were assessed in **f** (mean cell number, 162 ± 10). Data (mean ± SEM). Unpaired *t* test *p < 0.01, **p < 0.01. **g**, **h** mRNA expression of pro-inflammatory (**g**) and anti-inflammatory/immune-regulatory (**h**) markers in microglial cells sorted from P7 pups and cultured under basal and pro-inflammatory (IL-1β + INFγ) conditions ± LY 2389575 (5 μM). The data (mean ± SEM) are relative to the gene expression under basal CTRL conditions. One-way ANOVA followed by the Newman-Keuls multiple comparison test *p < 0.05, **p < 0.01, ***p < 0.001 effect of LY 2389575; ^$$^*p* < 0.01, ^$$$^*p* < 0.001, ^$$$$^*p* < 0.0001 effect of IL-1β + INFγ

According to optimal drug concentration (5 μM) conferring the highest effect on inflammatory-related gene expression in microglial cell culture, we next examined the effect of LY 2389575 treatment on the morphology of microglial cells sorted from CTRL pups at P4 and P7, with or without IL-1β + IFNγ challenge. Pharmacological blockade of mGlu3 receptors induced a change in cell morphology toward an amoeboid shape in both P4 and P7 microglia, with a significant reduction in cell area and perimeter and an increase in cell circularity (Fig. 4a, b, e, f).

We further characterized the effect of mGlu3 receptor blockade on microglial reactivity by studying the effect of LY 2389575, with or without IL-1β + IFNγ challenge, on the expression of inflammation-related markers in a second series of in vitro experiments. Pharmacological blockade of mGlu3 receptors increased the expression of all pro-inflammatory markers in primary microglia from P4 rat pups under basal conditions and in response to IL-1β + IFNγ (Fig. 4c). LY 2389575 also induced an increase in Mrc1 levels under basal conditions and a significant reduction of Arg1 expression in response to IL-1β + IFNγ (Fig. 4d). Similar effects were produced by LY 2389575 in microglia sorted from P7 pups (Fig. 4g). mGlu3 receptor blockade also reduced the expression of Il4ra under basal conditions and in response to the inflammatory challenge (Fig. 4h).

### Grm3 knock-out in mice and in vitro knock-down induce a pro-inflammatory phenotype in microglia

**Fig. 5:**
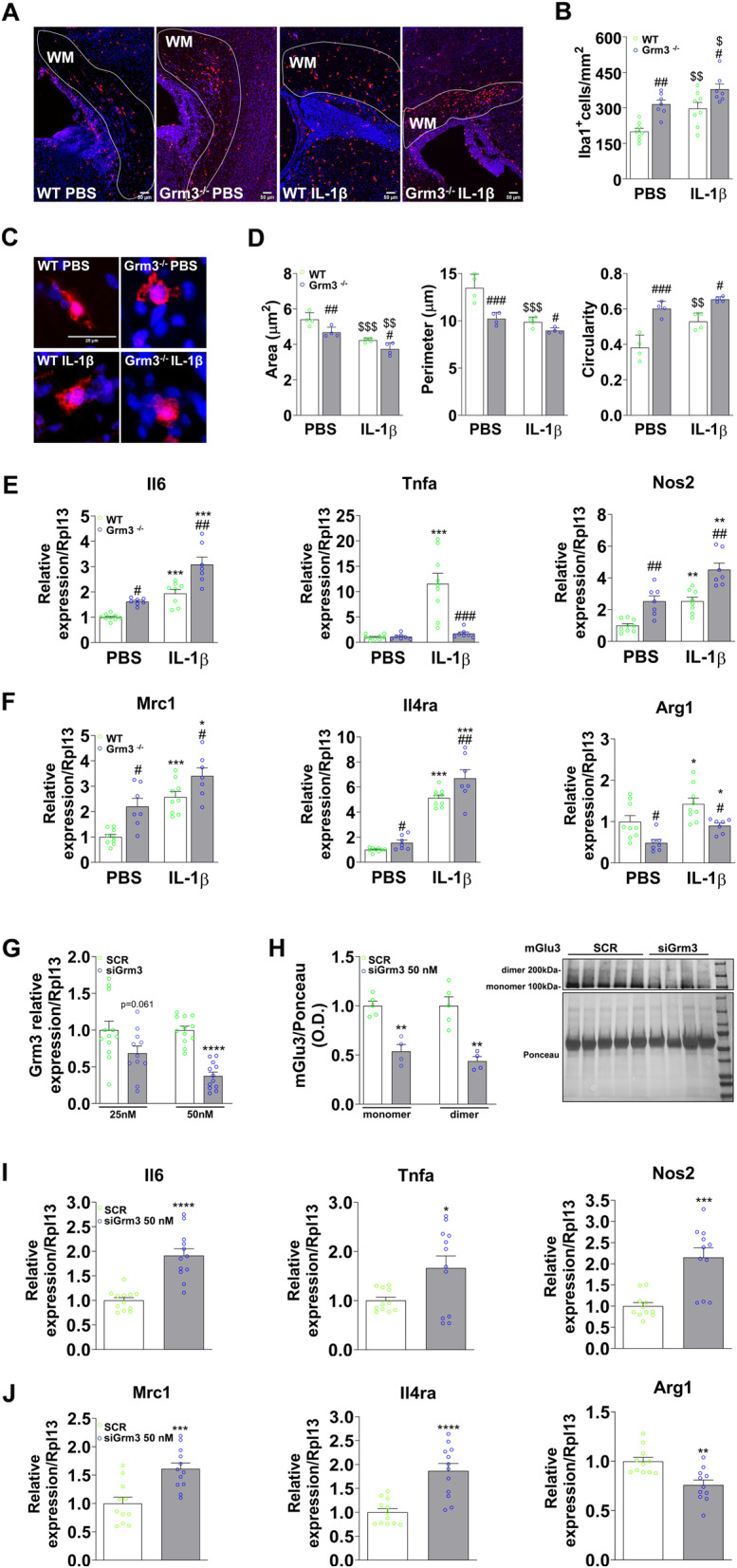
Effect of Grm3 genetic deletion in vivo and knock-down in vitro on microglial reactivity. **a**, **b** Iba1^+^ cells (red)/DAPI (blue) within Cingular white matter of P4 WT and Grm3^−/−^ mice under basal conditions and after IL-1β injections. Representative photomicrographs at × 10 magnification are shown in **a** (scale bar = 50 μm). Data (mean ± SEM) are expressed as number of Iba1^+^/mm^2^ in **b**. Two-way ANOVA followed by the Newman-Keuls multiple comparison; ^$^*p* < 0.05, ^$$^*p* < 0.01 effect of IL-1β + INFγ; ^#^*p* < 0.05, ^##^*p* < 0.01 effect of Grm3^−/−^. **c**, **d** Iba1^+^ cells within Cingular white matter of P4 WT and Grm3^−/−^ mice under basal conditions and after IL-1β injections. Representative photomicrographs at × 40 magnification are shown in **c** (scale bar = 25 μm). The cell area, cell perimeter, and cell circularity were assessed in **d**. Data (mean ± SEM). Two-way ANOVA followed by the Newman-Keuls multiple comparison; ^$$^*p* < 0.01, ^$$$^*p* < 0.001 effect of IL-1β + INFγ; ^#^*p* < 0.05, ^##^*p* < 0.01, ^###^*p* < 0.001 effect of Grm3^−/−^. **e**, **f** mRNA expression of pro-inflammatory (**e**) and anti-inflammatory/immune-regulatory (**f**) markers in Prefrontal/Cingular cortex isolated from P4 WT and Grm3^−/−^ mice under basal conditions and after IL-1β injections. Data (mean ± SEM) are relative to the mRNA expression of WT mice. Two-way ANOVA followed by the Newman-Keuls multiple comparison; *p < 0.05, **p < 0.01, ***p < 0.01 effect of IL-1β + INFγ; ^#^*p* < 0.05, ^##^*p* < 0.01, ^###^*p* < 0.001 effect of Grm3^−/−^. **g** Grm3 mRNA expression in primary microglia cultures 48 h after transfection with Grm3 siRNA (25 nM) and with Grm3 siRNA (50 nM). Data (mean ± SEM) are relative to Grm3 expression in the scramble group (SCR). Unpaired *t* test; ****p < 0.0001. **h** mGlu3 receptor protein expression in primary microglia cultures 48 h after transfection with Grm3 siRNA (50 nM). Data (mean ± SEM) are relative to mGlu3 receptor protein expression in the scramble group (SCR). Three wells per condition pooled. Unpaired *t* test; **p < 0.01. **i**, **j** mRNA expression of pro-inflammatory (**i**) and anti-inflammatory/immune-regulatory (**j**) markers in response to SCR and Grm3 siRNA (50 nM). Data (mean ± SEM) are relative to the mRNA expression for the respective SCR. Unpaired *t* test; *p < 0.05, **p < 0.01, ***p < 0.001, ****p < 0.0001

We analyzed the effect of Grm3 deletion on neuroinflammation in vivo. WT and Grm3^−/−^ mice were exposed to sub-threshold doses of IL-1β from P1 to P3. The density of Iba1^+^ cells in the Cingular white matter and the expression of inflammatory markers in the Prefrontal/Cingular cortex were analyzed at P4. Under basal conditions, Grm3 deletion induced a pro-inflammatory phenotype characterized by higher density of Iba1-positive cells within the Cingular white matter (Fig. 5a, b). These Iba1^+^ cells were characterized by an amoeboid shape, with a significant reduction in cell area and perimeter and an increase in cell circularity (Fig. 5c, d). As expected, the injection of IL-1β induced a significant increase in Iba1^+^ cell density in the Cingular white matter in both WT and Grm3^−/−^ animals (Fig. 5a, b) but to a larger extent in Grm3^−/−^ mice. These Iba1^+^ cells were also characterized by an amoeboid shape (Fig. 5c, d).

Grm3^−/−^ mice were characterized by a decreased expression of Tnfa and Arg1 gene and upregulation of Il6, Nos2, Mrc1, and Il4ra gene expression under basal conditions (Fig. 5e, f). IL-1β injections were associated with an increased expression of inflammatory markers in WT and Grm3^−/−^ animals (Fig. 5e, f). In Grm3^−/−^ mice, the increased expression of Il6, Nos2, Mrc1, and Il4ra induced by IL-1β exposure was exacerbated compared to WT.

We then used siRNA as a second strategy to knock-down mGlu3 receptor expression specifically in microglia sorted from CTRL pups. We first tested the transfection efficiency using siGLO Red (25 nM or 50 nM) obtaining a 58.2% (25 nM) and 83.2% (50 nM) transfection rate 48 h after the application of siGLO Red (Additional Fig 4A, B). Grm3 siRNA (25 nM) slightly reduced Grm3 expression (Fig. 5g) without modifying the level of inflammatory and anti-inflammatory cytokines’ gene expression (data not shown). In contrast, treatment with Grm3 siRNA (50 nM) significantly reduced mGlu3 receptor mRNA and protein levels after 48 h (Fig. 5g, h); increased the level of Il6, Nos2, Tnfa, Mrc1, and Il4ra transcripts; and reduced the level of the transcript for the anti-inflammatory cytokine Arg1 (Fig. 5i, j). Interestingly, the level of Grm3 expression was negatively correlated to the expression of Il6 (Pearson’s correlation: *r* = − 0.7572, *p* < 0.0001), Tnfa (*r* = − 0.5085, *p* = 0.0132), Nos2 (*r* = − 0.8115, *p* < 0.0001), Mrc1 (*r* = − 0.5302, *p* = 0.0093), and Il4ra (*r* = − 0.7956, *p* < 0.0001) and positively correlated to Arg1 expression (*r* = 0.5048, *p* = 0.0166) (Additional Fig 5A, B). Overall, these data strongly suggest that Grm3 ablation or knock-down induces a pro-inflammatory phenotype in microglia.

### mGlu3 receptor regulates Ccl9 chemokine expression in rat microglia

**Fig. 6:**
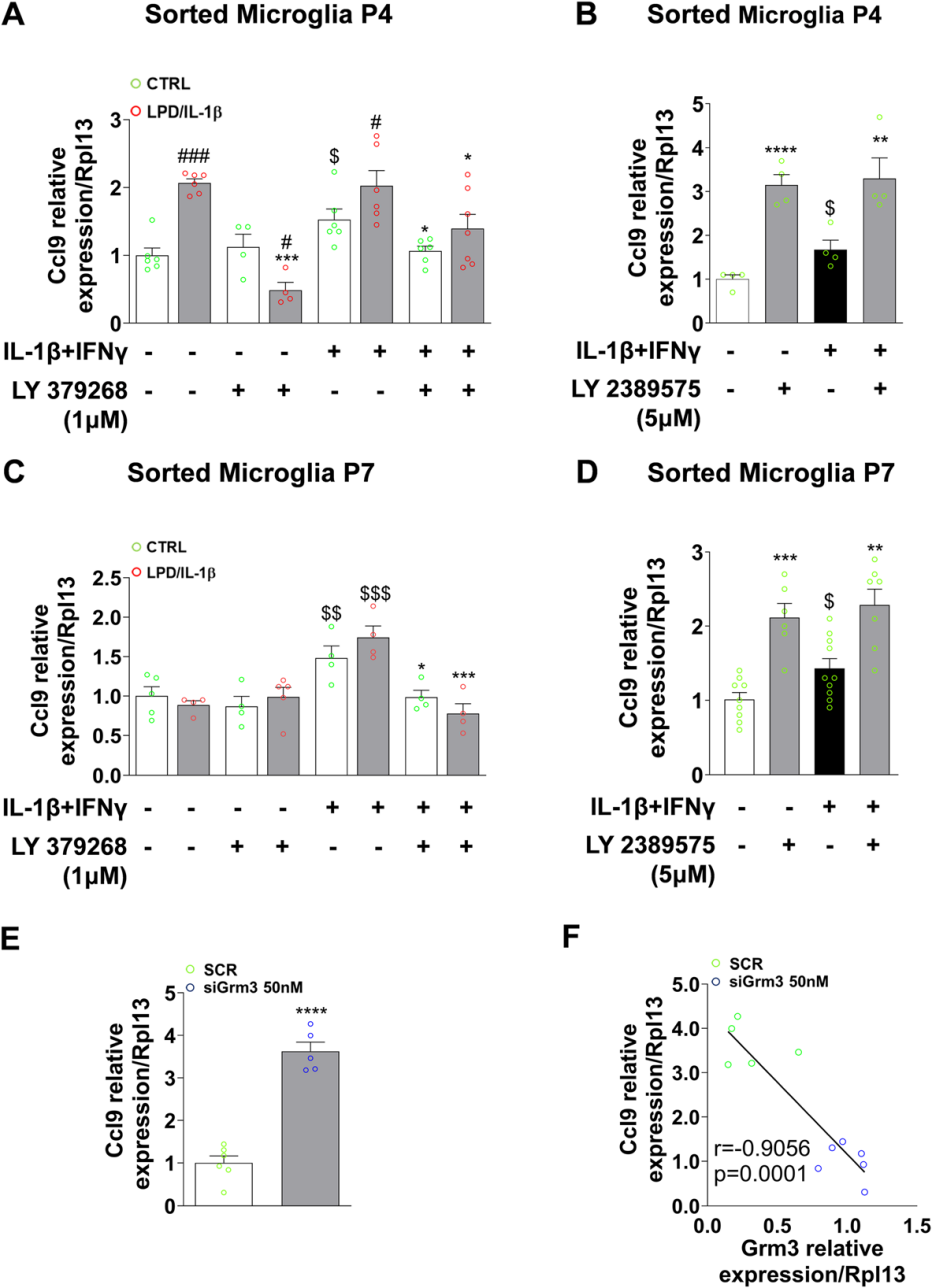
Effects of pharmacological mGlu3 receptor modulation and Grm3 knock-down on microglial Ccl9 expression in vitro. **a**, **c** mRNA expression of Ccl9 under the pro-inflammatory condition (IL-1β + IFNγ) in the presence of LY 379268 (1 μM) + Ro 64-5229 (25 μM) in CTRL and LPD/IL-1β cultured microglia at P4 (**a**) and at P7 (**c**). Data (mean ± SEM) are relative to the gene expression under basal CTRL conditions. Two-way ANOVA followed by the Newman-Keuls multiple comparison; *p < 0.05, ***p < 0.001 effect of LY 379268 + Ro 64-5229; ^$^*p* < 0.05, ^$$^*p* < 0.01, ^$$$^*p* < 0.001 effect of IL-1β + IFNγ; ^#^*p* < 0.05, ^###^*p* < 0.001 effect of LPD/IL-1β. **b**, **d** mRNA expression of Ccl9 in microglial cells sorted from P4 (**b**) and P7 (**d**) rat pups cultured under basal or pro-inflammatory (IL-1β + INFγ) conditions ± LY 2389575 (5 μM). The data (mean ± SEM) are relative to the gene expression under basal CTRL conditions. One-way ANOVA followed by the Newman-Keuls multiple comparison test; **p < 0.01, ***p < 0.001, ****p < 0.0001 effect of LY 2389575; ^$^*p* < 0.05 effect of IL-1β + INFγ. **e** Ccl9 mRNA expression in response to SCR and Grm3 siRNA (50 nM). Data (mean ± SEM) are relative to the mRNA expression for the respective SCR. Unpaired *t* test; ****p < 0.0001. **f** Pearson’s correlation between Grm3 expression and Ccl9 48 h after transfection with siGrm3 (50 nM)

Transcriptomic analyses of P4 LPD/IL-1β microglia highlighted the upregulation of Ccl9 gene expression, a chemokine included in the “Cytokine-cytokine receptor interaction/Chemokine signaling pathway,” a gene set highly connected to Grm3 network (Fig. 1f, g). We further studied Ccl9 expression under all in vitro conditions previously considered. P4 microglia sorted from LPD/IL-1β rats showed an increase of Ccl9 expression compared to CTRL both under basal and pro-inflammatory conditions (Fig. 6a). Pharmacological activation of mGlu3 receptors reversed Ccl9 overexpression observed in LPD/IL-1β microglia and reduced the effect of pro-inflammatory challenge on Ccl9 gene expression (Fig. 6a). In P7 microglia, mGlu3 receptors’ pharmacological activation was shown to induce Ccl9 gene downregulation only in pro-inflammatory conditions (Fig. 6c).

Pharmacological blockade of mGlu3 receptor by LY 2389575, with or without IL-1β + IFNγ challenge, was associated with Ccl9 upregulation both in P4 and P7 microglia (Fig. 6b, d).

Finally, siRNA-induced reduction of Grm3 gene expression increased Ccl9 transcripts (Fig. 6e), and a negative correlation between Grm3 gene expression and Ccl9 has also been observed (*r* = − 0.9056, *p* < 0.0001) (Fig. 6f).

## Discussion

We show that the mGlu3 receptor is highly expressed by microglia during early postnatal life and critically regulates microglial reactivity by exerting anti-inflammatory properties. We demonstrate that endogenous activation of microglia mGlu3 receptors limits inflammation and that they respond to pharmacological activation under pathological conditions, thus making them candidate drug targets for therapeutic intervention.

Prematurity and IUGR are associated with perinatal and postnatal inflammation, contributing to cerebral palsy and cognitive and behavioral disorders [1]. We developed a preclinical model of perinatal brain injury associated with IUGR by combining gestational LPD [37] with IL-1β injection of the pups. Animals exposed to this double-hit insult showed neuroinflammation and microglial increased activity, detected not only in vivo but also in cultured microglia sorted from the neonatal brain [32, 38].

The mGlu3 receptor is unique among all mGlu receptor subtypes because it was the only group II mGlu receptor downregulated in the microglia of pups exposed to LPD/IL-1β. Because pharmacological blockade or knock-down of mGlu3 receptors mimicked LPD/IL-1β inducing a pro-inflammatory microglial phenotype, it is reasonable to assume that the downregulation of mGlu3 receptors may contribute to neuroinflammation and the ensuing neurological complications associated with IUGR.

The age-dependent profile of Grm3 gene expression was not similar in the cerebral cortex and isolated microglia in rats. LPD/IL-1β reduced mGlu3 mRNA levels in the cerebral cortex at P4, but not at later postnatal days. In contrast, in microglia, mGlu3 receptor transcripts were found reduced at P4, P7, and P10, but not P20. The more limited downregulation of mGlu3 levels in the entire cerebral cortex may reflect the presence of the mGlu3 receptor in neurons and astrocytes [14], for which the impact of LPD/IL-1β on receptor expression is yet to be determined. A peak of microglial Grm3 gene expression was observed at P4–7 and a subsequent decline through to P20 in rats, but other single-cell RNAseq datasets in mice (http://www.microgliasinglecell.com) suggest that Grm3 microglial expression was similar at P4 and P30 and substantially increased at P100 [39]. These apparent discrepancies based on transcriptomic analyses between rats and mice may be explained by species specificity, difference in various sequencing depths, and the limitation of our experiments to the P20 developmental stage. Another explanation may originate from our microglia sorting method (MACS) with respect to the FACS used in the RNAseq experiments in mice.

We did not investigate the molecular mechanisms leading to mGlu3 downregulation here, but it is possible that there are transient epigenetic modifications and/or regulation of transcriptional factors that selectively and temporally change receptor expression in microglial cells exposed to LPD/IL-1β. This hypothesis is supported by an increased expression of the RE1-Silencing Transcription factor (REST) observed in LPD/IL-1β-exposed microglia cells. REST is a transcriptional repressor that silences target gene expression via epigenetic remodeling mechanisms [40]. Interestingly, it has been shown to bind to the promoter of Grm3 gene [41].

There are three distinct temporal stages in microglial development (“early microglia” until embryonic day 14, “pre-microglia” from embryonic day 14 to a few weeks after birth, and adult microglia afterwards). Transition through these stages, which is driven by a stepwise program of gene expression, critically regulates brain homeostasis [42]. Alterations of the microglial developmental trajectory during the perinatal period are associated with abnormal cell- and time-specific gene expression in other neural cell lineages, resulting in altered neuronal development [42, 43]. Early exposure to inflammation affects the transition between the maturational stages of microglia, causing a shift toward an advanced developmental stage [42, 44].

Glutamate is a key player in the functional crosstalk between microglia, neurons, and astrocytes, and microglial cells express both ionotropic and metabotropic glutamate receptors. The role of mGlu3 receptors in the modulation of microglial reactivity has been examined only in a few studies in relation to neurotoxicity associated with typical CNS disorders of adult life, such as Alzheimer’s disease and multiple sclerosis [15, 19, 22]. We were surprised to find that Grm3 was the major “hub” gene among several glutamate-related genes in the developing microglia and that it was the most mGlu receptor-encoding gene showing a robust response to an adverse perinatal environment. We activated mGlu3 receptors using the orthosteric agonist, LY 379268, which is brain permeant and displays a high affinity for mGlu3 receptors [45]. LY 379268 also has nanomolar affinity for mGlu2 receptors, but this bias was limited by the nearly absence of mGlu2 expression in the developing microglia (see Fig. 1) and by the use of Ro 64-5229, a negative allosteric modulator of mGlu2 receptors. Activation of mGlu3 receptors by LY 379268 reduced microglial activation in response to the in vitro inflammatory challenge and alleviated microglial higher activity following LPD/IL-1β exposure from birth to P7. Conversely, pharmacological blockade and knock-down of mGlu3 receptors enhanced microglial reactivity under both basal and activated conditions. Altogether, our findings show that mGlu3 receptors can modify microglia morphology and microglial expression of pro-inflammatory markers. They also suggest a close association between microglia morphology and function, as already reported [46–49].

These effects were also observed in vivo, both under basal and pro-inflammatory conditions in Grm3 KO mice. Because, periventricular white-matter injury is the most common cause of brain injury in preterm infants and the leading cause of neurocognitive disabilities [50], we primarily investigated the developing white matter in mice. This brain area is highly populated of microglial cells able to activate in response to systemic inflammation or any other brain insult [51]. Even if we studied an ablation of mGlu3 receptors not restricted to microglial cells, our findings are consistent with the hypothesis that mGlu3 receptors contribute to the regulation of microglia functions. Whether increase in inflammatory cytokines and chemokines observed in this KO may result from an increased cell proliferation or an increased gene expression in existing microglia alone remains to be elucidated. A notable exception regarding the microglia pro-inflammatory activation observed in Grm3 KO mice is the downregulation of Tnfa gene expression. This apparent discrepancy may be explained by the double-edge role of brain TNF-α in neuroinflammation modulation [52, 53] and opens a new perspective on the role of mGlu3 receptor in the regulation of TNF-α production.

The anti-inflammatory action of microglial mGlu3 receptors contrasts with the pro-inflammatory action of mGlu2 receptors found in various experimental models [19, 54]. mGlu3 and mGlu2 receptors share a similar amino acid sequence, pharmacological profile of activation, and transduction mechanisms. Both receptors are coupled to G_i/o_, and their activation inhibits adenylyl cyclase activity in heterologous expression systems [14]. We cannot exclude that the inhibition of cAMP formation drives the anti-inflammatory effects in the developing microglia, in which mGlu2 receptors were nearly absent. mGlu3 receptors are different from mGlu2 receptors in that they boost mGlu5 receptor signaling, enhancing mGlu5 receptor-mediated phospho-inositide hydrolysis. Crosstalk between mGlu3 and mGlu5 receptors has been demonstrated in heterologous expression systems, brain slice preparations, cultured astrocytes [29], and cultured microglia (authors’ unpublished observation).

A large body of evidence shows that mGlu5 receptor activation drives microglia toward an anti-inflammatory phenotype [20, 55–58] and restrains microglia-driven neuroinflammation in models of temporal lobe epilepsy [59], Parkinson’s disease [60], subarachnoid hemorrhage [61], and traumatic brain injury [62]. Indeed, a reduction of mGlu5 receptor expression in LPD/IL-1β microglia has been observed at P4 and in a lower extent at P7 and P10. It is therefore highly possible that the facilitation of mGlu5 receptor signaling is involved in the anti-inflammatory action of mGlu3 receptors in developing microglia. Functional crosstalk between mGlu3 receptors and G_q/11_-coupled oxytocin receptors has also been hypothesized to play a role in the anti-inflammatory effect of oxytocin [32]. Further studies using specific G protein inhibitors or co-stimulation of mGlu3 and other G protein-coupled receptors are needed to better understand the molecular determinants of the anti-inflammatory effects displayed by mGlu3 receptors in the developing microglia. The role of Grm3 in microglia should also be refined in further studies using a cell-specific KO. Indeed, the effect of the constitutive KO, as investigated here, could be an indirect effect and should be interpreted with caution.

## Conclusions

In conclusion, we show that the microglial mGlu3 receptor is highly downregulated in a rat model of perinatal brain injury and its pharmacological modulation a major determinant of microglial activity. The fine-tuning of microglial activation is key for preventing brain damage, because microglia not only are a cornerstone of the brain inflammatory response but also modulate certain functions of the brain environment, including synaptic pruning and maturation [63]. Selective pharmacological activation of mGlu3 receptors during early postnatal life might mitigate neuroinflammation associated with IUGR or other perinatal adverse conditions, thus preventing the development of neurological disorders later in life.

## Supplementary Information

**Additional file 1: Figure S1.** mGlu3 receptor agonist LY 379268 and microglial reactivity in response to inflammatory challenge: dose-response curve. (A, B) mRNA expression of pro-inflammatory (A) and anti-inflammatory/immune-regulatory (B) markers under the pro-inflammatory condition (IL-1β + IFNγ) in the presence of LY 379268 (0.1, 0.3, 1, 3, 5 μM) + Ro 64-5229 (25 μM). Data (mean ± SEM) are relative to the gene expression under basal CTRL conditions. One-way ANOVA followed by the Newman-Keuls multiple comparison; **p < 0.01, ***p < 0.001 effect of LY 379268 + Ro 64-5229; ^$$^*p* < 0.01, ^$$$^*p* < 0.001, ^$$$$^*p* < 0.0001 effect of IL-1β + IFNγ.

**Additional file 2: Figure S2.** mGlu3 receptor activation and microglial reactivity in CTRL and LPD/IL-1β cultured microglia at P7. (A, B) Microglial cells were stained with IBA1 (green) and DAPI (blue) under basal and challenged conditions (IL-1β + IFNγ) ± LY 379268 (1 μM) + Ro 64-5229 (25 μM). Representative photomicrographs at 40X magnification are shown in A (scale bar = 50 μm). Four cell-culture wells for each condition were analyzed in B (mean cell number: 155 ± 12) and the cell area, cell perimeter, and cell circularity were assessed. Data (mean ± SEM). Two-way ANOVA followed by the Newman-Keuls multiple comparison test; **p < 0.01, ***p < 0.001, ****p < 0.001 effect of LY 379268 + Ro 64-5229; ^$^*p* < 0.05, ^$$$^*p* < 0.001, ^$$$$^*p* < 0.001 effect of IL-1β + IFNγ; ^#^*p* < 0.05, ^##^*p* < 0.01, ^####^*p* < 0.0001 effect of LPD/IL-1β. (C, D) mRNA expression of pro-inflammatory (C) and anti-inflammatory/immune-regulatory (D) markers under basal and pro-inflammatory conditions ± LY 379268 (1 μM) + Ro 64-5229 (25 μM). Data (mean ± SEM) are relative to the gene expression under basal CTRL conditions. Two-way ANOVA followed by the Newman-Keuls multiple comparison test; *p* < 0.0001; *p < 0.05, **p < 0.01, ***p < 0.001, ****p < 0.0001 effect of LY 379268+ Ro 64-5229; ^#^*p* < 0.05, ^##^*p* < 0.01, ^###^*p* < 0.001, effect of LPD/IL-1β; ^$$$$^*p* < 0.0001 effect of IL-1β + IFNγ.

**Additional file 3: Figure S3.** Pharmacological mGlu3 receptor blockade and microglial reactivity in response to inflammatory stimulation: dose-response curve. (A, B) mRNA expression of pro-inflammatory (A) and anti-inflammatory/immunoregulatory (B) markers under the pro-inflammatory condition (IL-1β + IFNγ) in the presence of the mGlu3 negative allosteric modulator LY 2389575 (0.1, 0.3, 1, 3, 5 μM). Data (mean ± SEM) are relative to the gene expression under basal CTRL conditions. One-way ANOVA followed by the Newman-Keuls multiple comparison test; *p < 0.05, **p < 0.01, ***p < 0.001, effect of LY 2389575; ^$^*p* < 0.05, ^$$^*p* < 0.01, ^$$$^*p* < 0.001, ^$$$$^*p* < 0.0001, effect of IL-1β + IFNγ.

**Additional file 4: Figure S4.** Transfection efficiency in rat primary cultured microglia. (A, B) Microglial cells sorted from P7 CTRL rat pups were stained with Iba1 (green) and DAPI (blue) 48h after transfection with siGLO Red Transfection indicator (25nM and 50nM). Representative photomicrographs at 10X magnification are shown in A (scale bar = 50 μm). Value are relative to the percentage of microglia siGLO Red^+^cells/DAPI in B. Data (mean ± SEM); four culture wells per condition (mean cells number 25nM: 141 ± 12; mean cells number 50nM: 165 ± 9). Unpaired *t*-test; ***p < 0.001.

**Additional file 5: Figure S5.** Pearson correlation between Grm3 expression and inflammatory markers 48h after siGrm3 transfection. (A, B) Pearson correlation between Grm3 expression and pro-inflammatory markers (A), and between Grm3 expression and anti-inflammatory/immune-regulatory markers (B), 48h after transfection with siGrm3 (50nM).

**Additional file 6: Table S1.** Number of samples, statistics, and *p* values.

## Data Availability

Raw data reported in the manuscript are archived at the EMBL-European Bioinformatics Institute (EBI) under E-MATB-6631.

## Abbreviations

CNS: Central nervous system
CTRL: Control
IUGR: Intra-uterine growth restriction
KO: Knock-out
LPD: Low-protein diet
MACS: Magnetic antibody-based cell sorting
mGlu3 receptor: Metabotropic glutamate receptor 3
WT: Wild type
