# Supplementary figures and images for: mGlu3 receptor regulates microglial cell reactivity in neonatal rats
Source: J Neuroinflammation. 2021 Jan 6;18:13. doi: 10.1186/s12974-020-02049-z (PMC7789385; doi:10.1186/s12974-020-02049-z)

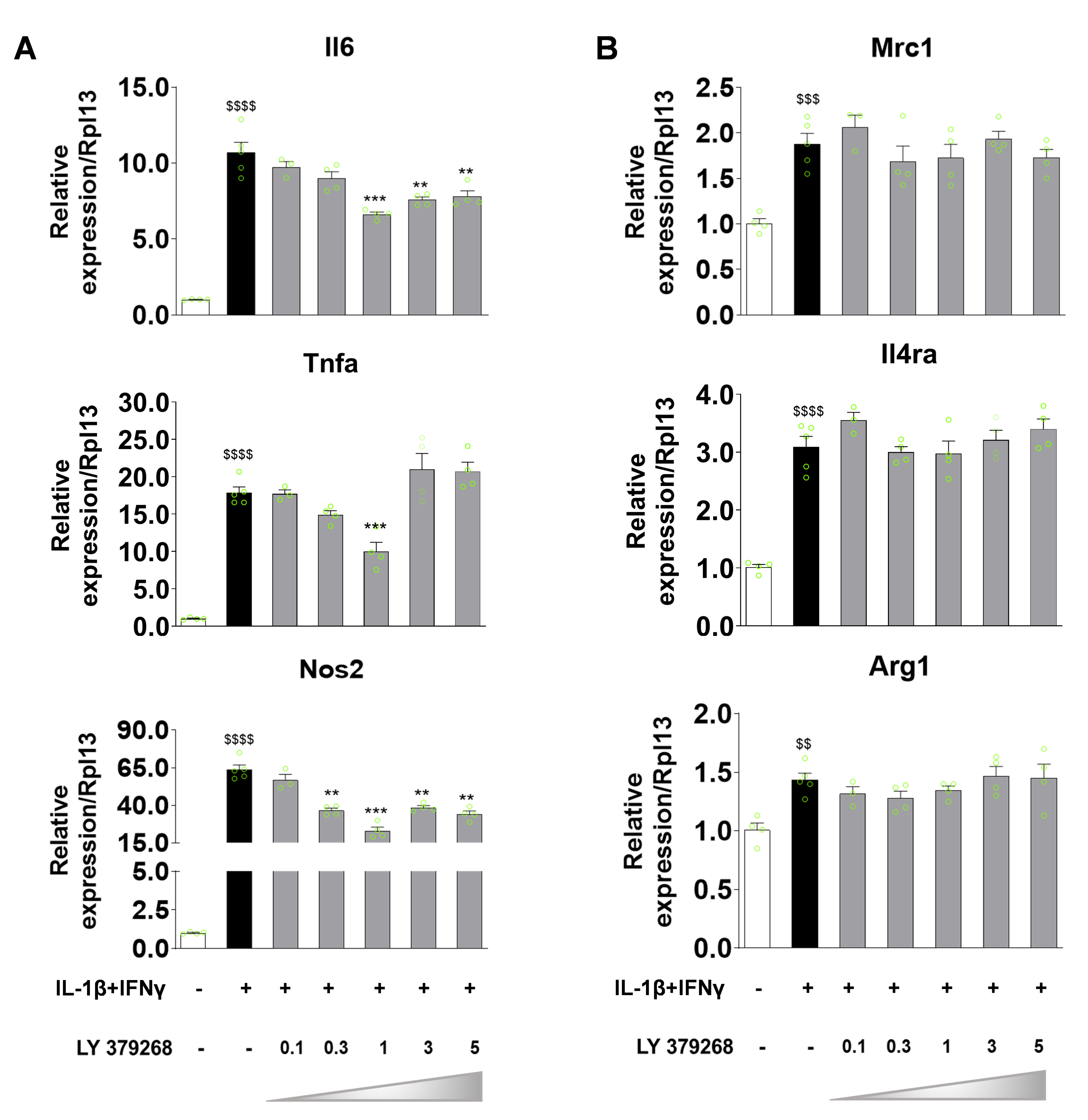

Supplement: Supplementary file 1 — Additional file 1: Figure S1. mGlu3 receptor agonist LY 379268 and microglial reactivity in response to inflammatory challenge: dose-response curve. (A, B) mRNA expression of pro-inflammatory (A) and anti-inflammatory/immune-regulatory (B) markers under the pro-inflammatory condition (IL-1β + IFNγ) in the presence of LY 379268 (0.1, 0.3, 1, 3, 5 μM) + Ro 64-5229 (25 μM). Data (mean ± SEM) are relative to the gene expression under basal CTRL conditions. One-way ANOVA followed by the Newman-Keuls multiple comparison; **p < 0.01, ***p < 0.001 effect of LY 379268 + Ro 64-5229; $$p < 0.01, $$$p < 0.001, $$$$p < 0.0001 effect of IL-1β + IFNγ. [file 12974_2020_2049_MOESM1_ESM.png]

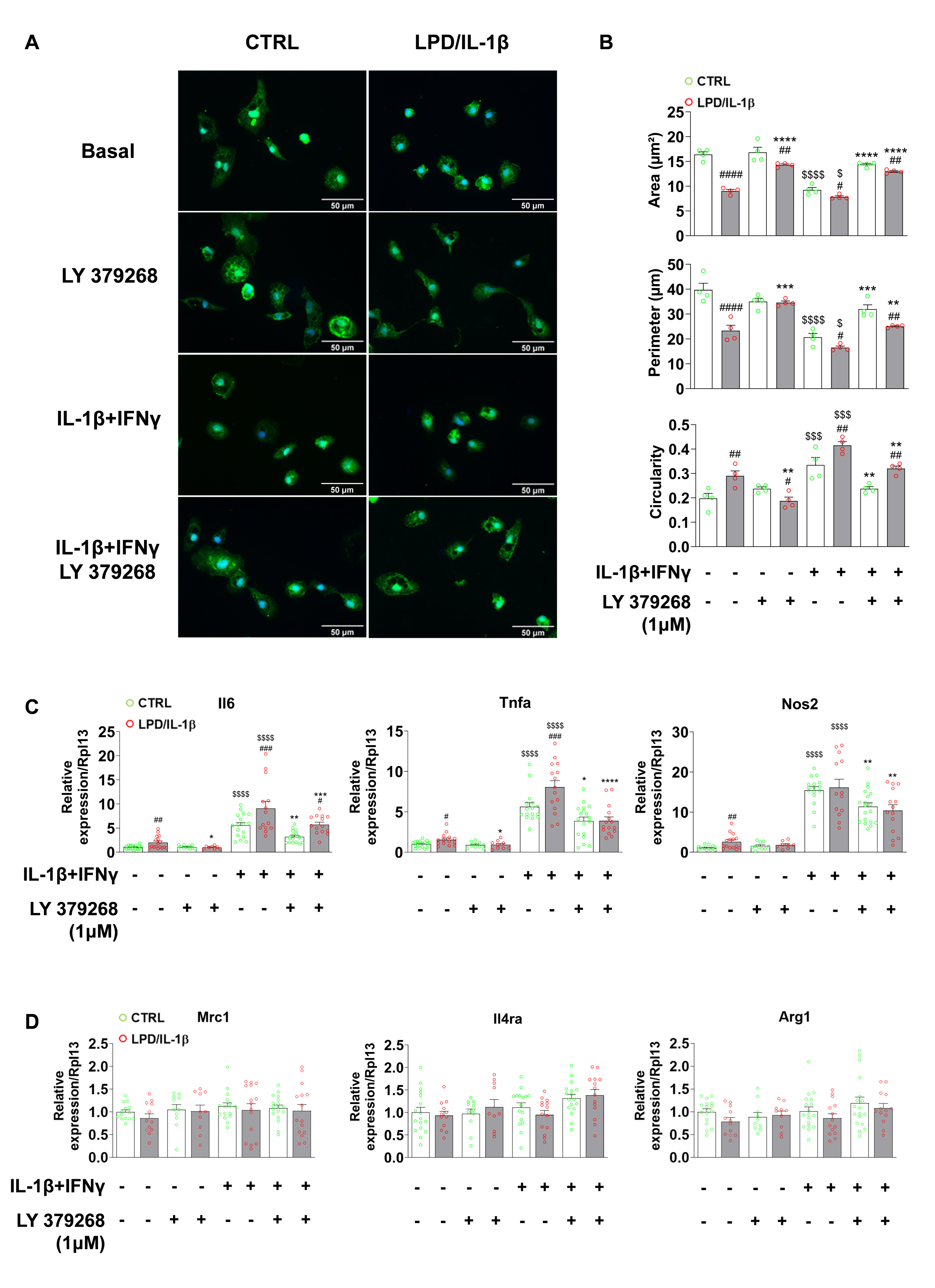

Supplement: Supplementary file 2 — Additional file 2: Figure S2. mGlu3 receptor activation and microglial reactivity in CTRL and LPD/IL-1β cultured microglia at P7. (A, B) Microglial cells were stained with IBA1 (green) and DAPI (blue) under basal and challenged conditions (IL-1β + IFNγ) ± LY 379268 (1 μM) + Ro 64-5229 (25 μM). Representative photomicrographs at 40X magnification are shown in A (scale bar = 50 μm). Four cell-culture wells for each condition were analyzed in B (mean cell number: 155 ± 12) and the cell area, cell perimeter, and cell circularity were assessed. Data (mean ± SEM). Two-way ANOVA followed by the Newman-Keuls multiple comparison test; **p < 0.01, ***p < 0.001, ****p < 0.001 effect of LY 379268 + Ro 64-5229; $p < 0.05, $$$p < 0.001, $$$$p < 0.001 effect of IL-1β + IFNγ; #p < 0.05, ##p < 0.01, ####p < 0.0001 effect of LPD/IL-1β. (C, D) mRNA expression of pro-inflammatory (C) and anti-inflammatory/immune-regulatory (D) markers under basal and pro-inflammatory conditions ± LY 379268 (1 μM) + Ro 64-5229 (25 μM). Data (mean ± SEM) are relative to the gene expression under basal CTRL conditions. Two-way ANOVA followed by the Newman-Keuls multiple comparison test; p < 0.0001; *p < 0.05, **p < 0.01, ***p < 0.001, ****p < 0.0001 effect of LY 379268+ Ro 64-5229; #p < 0.05, ##p < 0.01, ###p < 0.001, effect of LPD/IL-1β; $$$$p < 0.0001 effect of IL-1β + IFNγ. [file 12974_2020_2049_MOESM2_ESM.png]

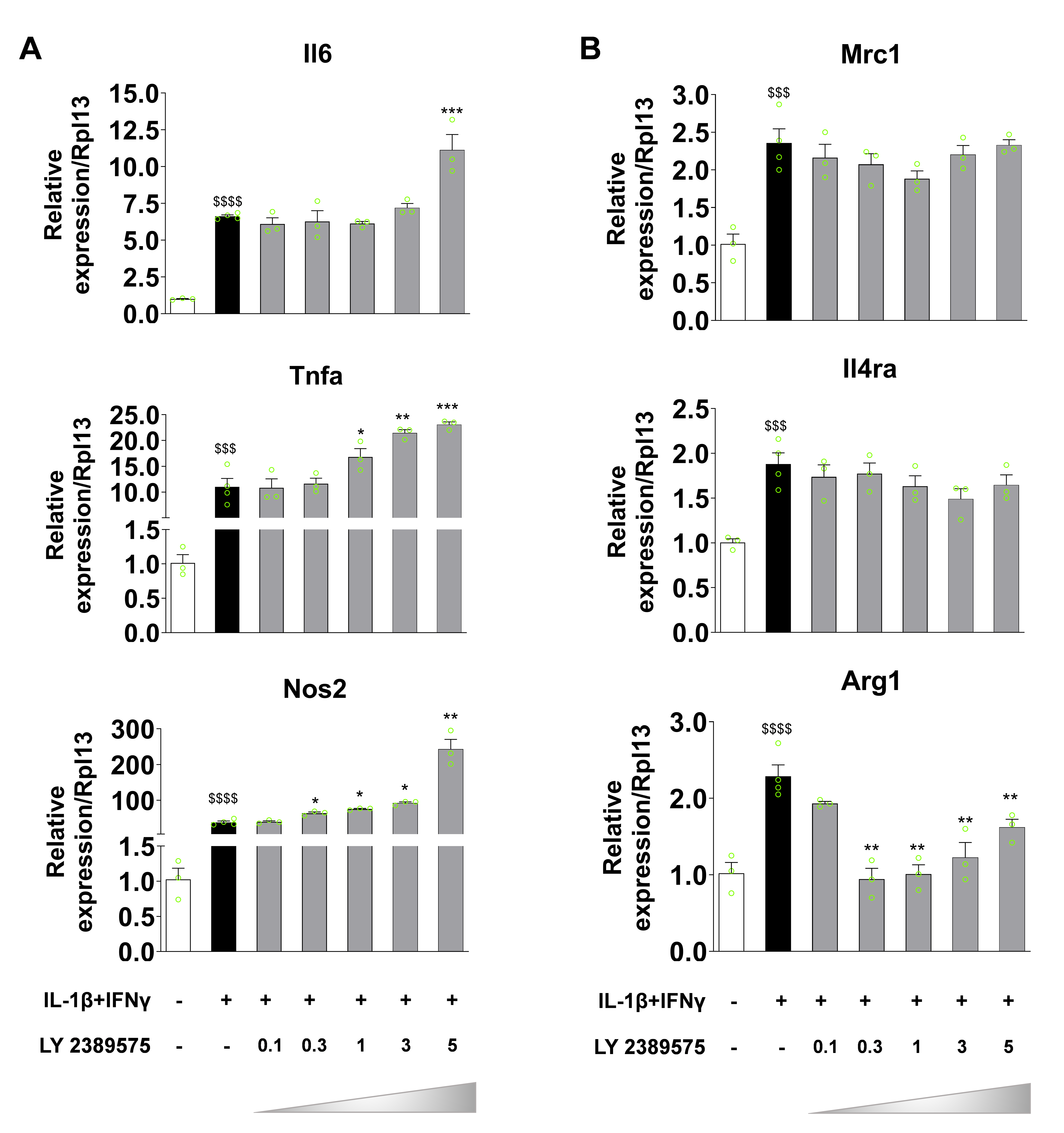

Supplement: Supplementary file 3 — Additional file 3: Figure S3. Pharmacological mGlu3 receptor blockade and microglial reactivity in response to inflammatory stimulation: dose-response curve. (A, B) mRNA expression of pro-inflammatory (A) and anti-inflammatory/immunoregulatory (B) markers under the pro-inflammatory condition (IL-1β + IFNγ) in the presence of the mGlu3 negative allosteric modulator LY 2389575 (0.1, 0.3, 1, 3, 5 μM). Data (mean ± SEM) are relative to the gene expression under basal CTRL conditions. One-way ANOVA followed by the Newman-Keuls multiple comparison test; *p < 0.05, **p < 0.01, ***p < 0.001, effect of LY 2389575; $p < 0.05, $$p < 0.01, $$$p < 0.001, $$$$p < 0.0001, effect of IL-1β + IFNγ. [file 12974_2020_2049_MOESM3_ESM.png]

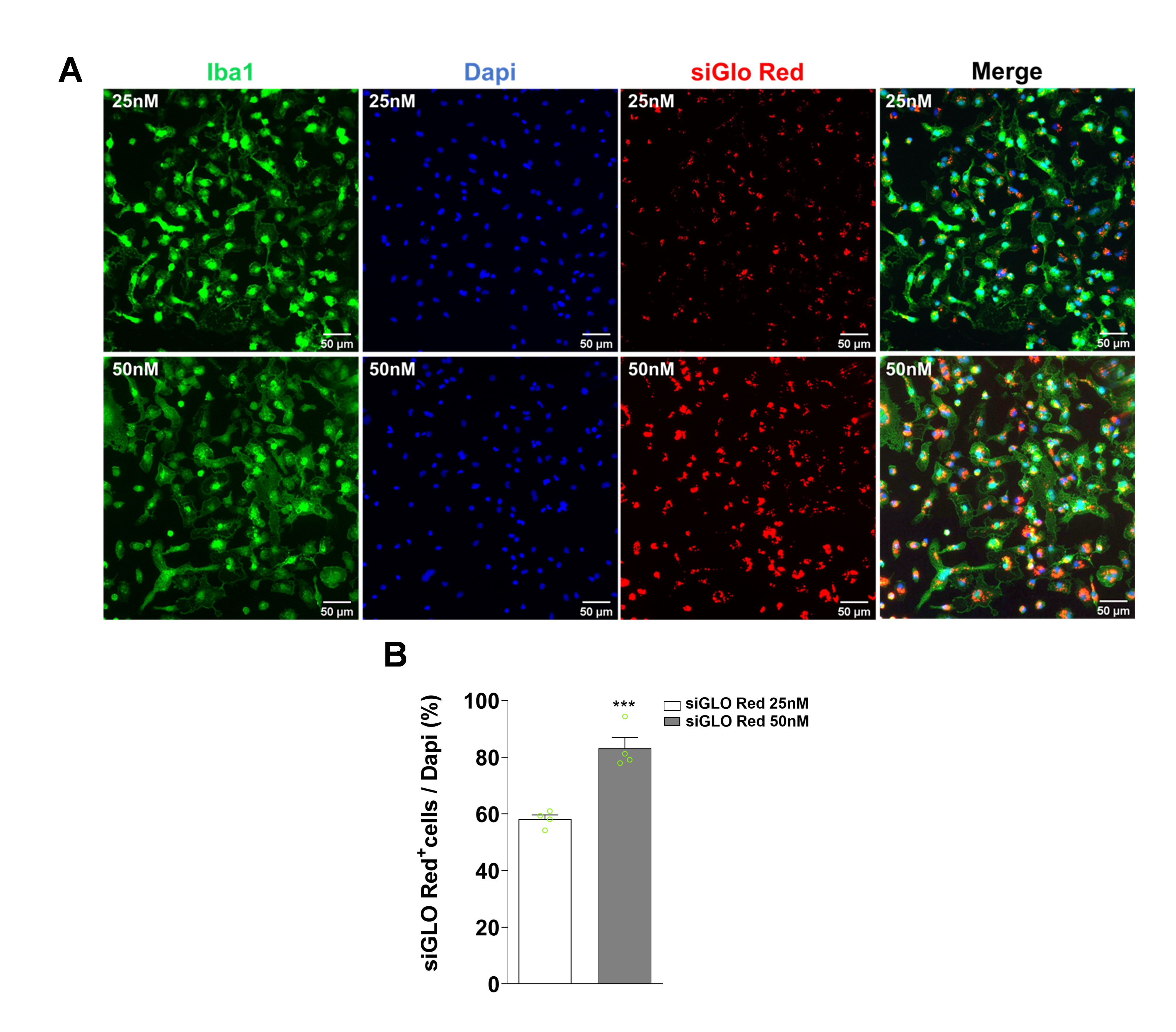

Supplement: Supplementary file 4 — Additional file 4: Figure S4. Transfection efficiency in rat primary cultured microglia. (A, B) Microglial cells sorted from P7 CTRL rat pups were stained with Iba1 (green) and DAPI (blue) 48h after transfection with siGLO Red Transfection indicator (25nM and 50nM). Representative photomicrographs at 10X magnification are shown in A (scale bar = 50 μm). Value are relative to the percentage of microglia siGLO Red+cells/DAPI in B. Data (mean ± SEM); four culture wells per condition (mean cells number 25nM: 141 ± 12; mean cells number 50nM: 165 ± 9). Unpaired t-test; ***p < 0.001. [file 12974_2020_2049_MOESM4_ESM.png]

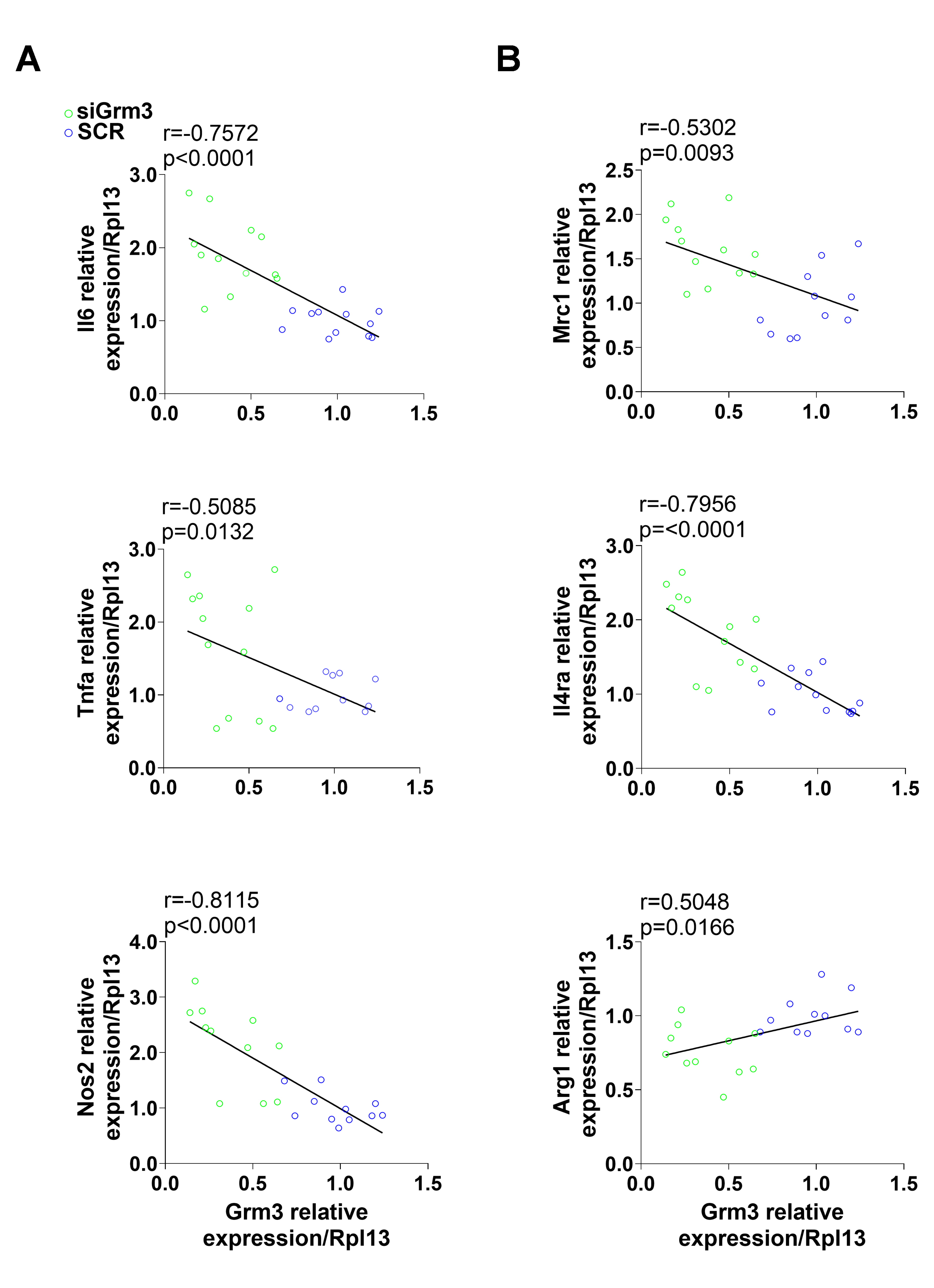

Supplement: Supplementary file 5 — Additional file 5: Figure S5. Pearson correlation between Grm3 expression and inflammatory markers 48h after siGrm3 transfection. (A, B) Pearson correlation between Grm3 expression and pro-inflammatory markers (A), and between Grm3 expression and anti-inflammatory/immune-regulatory markers (B), 48h after transfection with siGrm3 (50nM). [file 12974_2020_2049_MOESM5_ESM.png]
